# A235 INFLAMMATORY BOWEL DISEASE AND PREECLAMPSIA: A PHYSICIAN SURVEY

**DOI:** 10.1093/jcag/gwad061.235

**Published:** 2024-02-14

**Authors:** Y Hanna, P Tandon, V Huang

**Affiliations:** Department of General Internal Medicine , University of Toronto, Toronto, ON, Canada; University Health Network, Toronto, ON, Canada; Department of General Internal Medicine , University of Toronto, Toronto, ON, Canada

## Abstract

**Background:**

Preeclampsia is a disorder of pregnancy recognized as the second cause of maternal mortality worldwide. The association between IBD and preeclampsia remains unclear, but the significant comorbid profile, and lack of treatment aside from urgent delivery, make primary prevention a priority.

Aspirin was shown to halve preeclampsia rates and is recommended for primary prevention for women at risk. Despite high-quality evidence supporting Aspirin use and its well tolerated side effect profile, studies in non-IBD patients have demonstrated a knowledge-to-practice gap.

**Aims:**

While there are no studies reported in women with IBD, prescribing rates may be even lower in this population, given that it may be incorrectly perceived to increase the risk of an IBD flare. Understanding physician perceptions could allow for a targeted educational approach to increase patient and physician awareness of the indications for Aspirin prophylaxis and its safety profile.

**Methods:**

This is a cross-sectional survey study assessing physician perceptions and Aspirin prescribing patterns for preeclampsia prevention conducted from Mount Sinai Hospital. Demographic information, information on perceptions of preeclampsia risk in pregnant women with IBD, and on the perceived clinical benefit and risk of Aspirin prescribing were collected.

**Results:**

A total of 38 Canadian healthcare professionals (HCPs) (15 Gastroenterologists, 14 Obstetricians, 5 General Practitioners, 2 General Internists, 1 nurse practitioner, and 1 midwife) were surveyed. Most HCPs were practicing for over 10 years (71%). In total, 68% of HCPs were comfortable with pregnancy-specific management of IBD. Most HCPs correctly identified all comorbidities associated with a high risk of preeclampsia including a history of preeclampsia (95%), renal disease (87%), and autoimmune disease (68%). A total of 55% of HCPs believed that pregnant patients with IBD were at increased risk of placental related diseases, and 56% agreed that these patients were at increased risk of preeclampsia specifically. Thirty-five percent of HCPs believed that IBD in remission, in the absence of other risk factors, was an indication for Aspirin prophylaxis. More so, 45% believed that IBD, with poor disease control, was an indication.

Only 8% of HCPs believed that Aspirin, when used for preeclampsia prevention, was associated with an IBD flare. In patients whom Aspirin prophylaxis was indicated, 77% agreed with its use, even in patients in whom disease control was poor. Finally, 63% agreed that the Aspirin dose should be 162 mg daily, and more than half (58%) agreed that the prescriber should be the patient’s obstetrician.

**Conclusions:**

Most HCPs agreed that IBD was a risk factor for preeclampsia and that Aspirin prophylaxis was effective and safe for primary prevention. High quality studies are needed to evaluate risk of preeclampsia in IBD, especially in patients with active disease.

**Funding Agencies:**

CAG

